# ﻿*Liliumbrunneum* (Liliaceae): a newly discovered species in north-western Yunnan, China

**DOI:** 10.3897/phytokeys.254.147769

**Published:** 2025-04-03

**Authors:** Ting Wang, Xiuying Shen, De Wang, Ying Zhao, Xiaomei Qu, Yundong Gao

**Affiliations:** 1 CAS Key Laboratory of Mountain Ecological Restoration and Bioresource Utilization & Ecological Restoration and Biodiversity Conservation Key Laboratory of Sichuan Province, Chengdu Institute of Biology, Chinese Academy of Sciences, Chengdu 610213, China Chengdu Institute of Biology, Chinese Academy of Sciences Chengdu China; 2 University of Chinese Academy of Sciences, Beijing 100049, China University of Chinese Academy of Sciences Beijing China; 3 Fugong Administrative Sub-Bureau of Gaoligongshan National Nature Reserve, Nujiang Prefecture, Nujiang 673499, China Fugong Administrative Sub-Bureau of Gaoligongshan National Nature Reserve Nujiang China

**Keywords:** Hengduan Mountains, Liliaceae, *Liliumbrunneum*, *Liliumsouliei* complex, new species

## Abstract

*Liliumbrunneum* represents a newly discovered and described lily species with a limited distribution in isolated alpine regions of north-western Yunnan, China. The recognition of this new species as a distinct entity is supported by both morphological and molecular data. Furthermore, the investigation of this region, identified as a ‘hotspot’ due to its high diversity and endemicity within the genus *Lilium*, is anticipated to provide greater insight into the processes of speciation and the maintenance of species boundaries in this genus. Further fieldwork aimed at exploring these regions is expected to discover additional new species and therefore warrants special attention and resources.

## ﻿Introduction

The southwestern region of China, which encompasses the Hengduan Mountains and the Tibetan Plateau, is renowned for its numerous mountain ranges. Amongst these, the Hengduan Mountains and Eastern Himalaya Mountain ranges are recognised as two of the world’s biodiversity hotspots (Marchese 2015; Yu et al. 2020). Over an extended period, beginning in the late Miocene, the Hengduan Mountains experienced complex orogeny, notably characterised by rapid uplift, resulting in a highly rugged terrain and significant environmental heterogeneity (Wang et al. 2012). This diverse ecological landscape has led to the region being described as a cradle of evolution due to its remarkable biodiversity (Lu et al. 2018). While these mountainous regions provide favorable conditions for the survival of wildlife, their remote and rugged nature poses challenges to exploration efforts. The extensive expanse of unexplored regions presents opportunities for discovering previously unidentified or unrecognised species, largely due to a lack of investigation and sampling.

The genus *Lilium* L., a prominent member of the monocot family Liliaceae, comprises approximately 123 species (POWO 2024; WFO 2025). This genus consists of non-climbing, bulbous herbaceous plants (Peruzzi 2016) and is distributed across mid to high latitudes of the Northern Hemisphere (Liang and Tamura 2000; Skinner 2002). Extant species are primarily concentrated in temperate regions of the Northern Hemisphere, while East Asia hosts a notably greater diversity of *Lilium* species compared to Central Asia, Europe and North America (Gong et al. 2017; Dhiman et al. 2020). The Hengduan Mountains and the Himalayas are considered to be the central distribution area of *Lilium* (De Jong 1974; Du et al. 2014, 2017). However, the taxonomic status of several species in the Hengduan Mountain region remains unclear and requires clarification.

In our previous studies focusing on this region – particularly upon members of the former genus *Nomocharis* Franchet – we discovered that campanulate-flowered species, such as *Liliumsouliei* (Franch.) Sealy, and its close relatives, are nested within the Nomocharis-clade (Gao et al. 2013b, 2015; Yuan and Gao 2024). This finding reveals unexpected relationships given the significant differences in phenotypic characteristics. These alpine lilies, characterised by their dwarf habit and bell-shaped, nodding flowers, include *L.souliei*, *L.saccatum* S.Yun Liang, *L.medogense* S.Yun Liang, *L.paradoxum* Stearn and *L.georgei* (W.E. Evans) Sealy. Several of these species were only described in the last century, with few records existing other than the type specimens, resulting in potential taxonomic confusion within this group. Consequently, we will refer to this group as the *L.souliei* complex for brevity.

**Figure 1.**
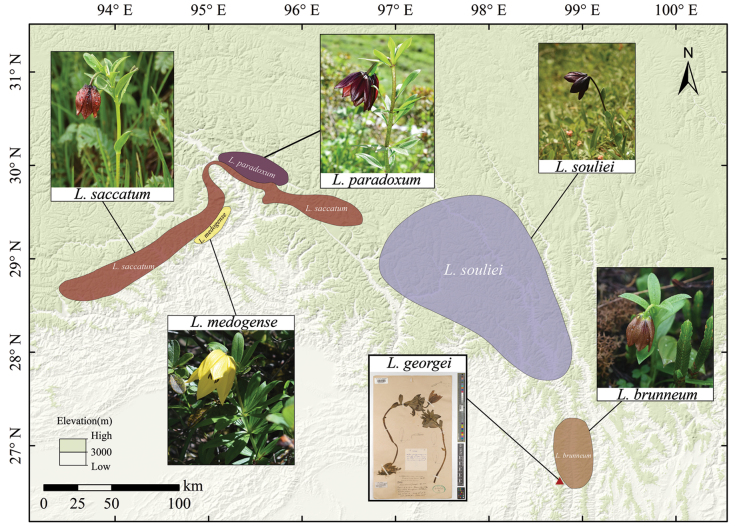
Morphological characteristics and geographic distribution of the *Liliumsouliei* complex. (*L.georgei*, http://specimens.kew.org/herbarium/K003949823).

According to field records accumulated and verified by the authors, the *L.souliei* complex is distributed from north-western Yunnan Province to south-eastern Tibet, traversing the Hengduan and extending into the Eastern Himalaya Mountain ranges (Dhiman et al. 2020). Amongst these, *L.souliei* has the broadest distribution, thriving in alpine grass and shrub habitats at elevations of 2800–4000 m above sea level (Wu et al. 2012), primarily in the central part of the Hengduan Mountains. *L.saccatum* is predominantly found in the shrub and grassland slopes of the Eastern Himalaya at approximately 3900 m (Liang 1987). In contrast, *L.paradoxum* and *L.medogense* exhibit much narrower distributions. *L.paradoxum* is endemic to southeastern Tibet, with limited populations at around 3500 m (Stearn 1956), while *L.medogense* is confined to alpine wetland habitats around 3600 m (Liang 1985) in Motuo (Medog) County, Xizang, China (Fig. 1).

With further investigations in the Hengduan Mountains and surrounding areas, coupled with advancements in molecular phylogenetics, we recognise that this is an opportune time to clarify the status of several monophyletic yet taxonomically ambiguous lily groups in the region. In this context, the *L.souliei* complex serves as an exemplary focus for detailed investigation and taxonomic revision. Through a comprehensive review of the literature and examination of herbarium specimens, the author, Gao, proposes that *L.georgei* (W.E. Evans) Sealy, first discovered by George Forrest in 1924 along the Myanmar-China border (Sealy 1950), also belongs to this complex.

In the summers of 2023 and 2024, the authors undertook two consecutive field trips to the remote areas of the Gaoligong Mountains in Yunnan (mid to southern part of the Hengduan Mountains), aiming to rediscover this lily and collect additional information and materials since no records exist beyond those documented by Forrest. Unfortunately, *L.georgei* was not located, as access to the type locality is currently restricted; however, a new species belonging to this group was discovered and is described in this paper.

In the present study, we utilise both morphological and molecular data to elucidate the status and phylogenetic position of the putative new species by comparing its morphology with that of closely related taxa. Simultaneously, this study aims to provide a more informative overview of the *L.souliei* complex, highlighting distinguishing characteristics amongst species and clarifying geographic distribution patterns. We hope this research will illuminate the diversity of lilies in the Hengduan Mountains and adjacent areas, contributing to and enriching the catalogue of the genus *Lilium*.

## ﻿Materials and methods

### ﻿Field sampling

The new species was observed and photographed exclusively in two locations: Fugong County, Yunnan, China, in the Gaoligong Mountains (on the border with Myanmar) and near Zhiziluo in the Biluoxueshan Range; and material was collected for herbarium specimens and molecular study. At these locations, mature individuals were counted, and the extent of their distribution was estimated to support a conservation assessment using GeoCAT software (Bachman et al. 2011) and IUCN criteria. The collected images and specimens were subsequently used for measurements and descriptions. Voucher specimens have been deposited in the herbarium of the Chengdu Institute of Biology (**CDBI**, Fig. 5A). Fresh leaves were collected and rapidly dried using silica gel.

### ﻿Morphological analysis

A comprehensive review of the relevant taxa was conducted through online databases, including Tropicos (https://tropicos.org/) and the Biodiversity Heritage Library (BHL, https://www.biodiversitylibrary.org/). Specimens were accessed through both physical and online herbarium collections, including the CDBI, E, K, KUN, IBSC, P, PE and SZ herbaria (acronyms according to Thiers 2024); the Chinese Virtual Herbarium (https://www.cvh.ac.cn/), the Kew Herbarium Catalogue (http://apps.kew.org/herbcat/gotoHomePage.do) and JSTOR Global Plants (https://plants.jstor.org/). This methodology was designed to facilitate a morphological comparison analysis based on a large and diverse set of specimens. The morphological traits selected for analysis were informed by key taxonomic features outlined in the Flora of China (Liang and Tamura 2000), including characteristics of the bulb, stem, leaf and flower. Eighteen morphological traits of the new species and its closely related taxa were measured using MATO (Liu et al. 2023). A list of morphological characters and their acronyms used in analyses is shown in (Suppl. material 1: table S1).

Principal component analysis (PCA) was performed on the standardised data for 18 quantitative traits (Suppl. material 1) using the built-in prcomp() function in R version 4.3.1 to achieve dimensionality reduction and feature extraction. The factoextra (Kassambara and Mundt 2017) and ggplot2 (Wickham et al. 2016) packages were installed and the fviz_pca_biplot() function was used to generate a biplot combining principal components and variables.

### ﻿Molecular phylogeny inference

Genomic DNA was extracted from silica-gel dried leaves using a modified cetyltrimethylammonium bromide (CTAB) method (Allen et al. 2006). Paired-end sequencing libraries were then constructed with insert sizes of approximately 350 bp, followed by sequencing on the DNBSEQ-T7 platform (Beijing Genomics Institute, BGI), with a depth of about 0.1 ~ 0.2 × (10G pair ending reads). Approximately 10–12 Gb of raw data were filtered and evaluated using fastp v0.23.2 (Chen et al. 2018) and FastQC (https://www.bioinformatics.babraham.ac.uk/projects/fastqc/) with default parameters. Chloroplast and Internal Transcribed Spacer (ITS) sequences were assembled from the clean data using GetOrganelle v1.7.6.1 (Jin et al. 2020). Chloroplast sequences were selected with the correct orientation through multiple sequence alignment using Mafft v7 (https://mafft.cbrc.jp/alignment/software/, (Katoh et al. 2019)) and the chloroplast genome was annotated using Geneious Prime v2023.1.2 (Biomatters Ltd, Auckland, New Zealand).

To infer the phylogenetic position of the newly described species, newly generated DNA sequences were combined with publicly available sequences, including thirty-four ITS and thirty-three chloroplast genomes from NCBI (https://www.ncbi.nlm.nih.gov/). Based on previous research (Yuan and Gao 2024), we selected the majority of species from the Nomocharis-clade (In particular, as many individuals as possible of *Liliumsouliei*, *L.saccatum*, *L.medogense* and *L.paradoxum*), as well as representative species from 2–3 closely related clades. The outgroup included three species from *Fritillaria*, *Cardiocrinum* and *Notholirion* (Suppl. material 2: table S2). Whenever possible, the ITS and chloroplast sequences used for phylogenetic analysis were derived from the same individual.

Maximum Likelihood (ML) analyses of both chloroplast and ITS data were conducted using an online platform (https://ngphylogeny.fr/; Lemoine et al. 2019). Sequences were analysed with an advanced workflow utilising the PhyML + SMS/OneClick method. The MAFFT, BMGE and PhyML + SMS workflow (for Maximum Likelihood phylogenetic tree inference based on smart model selection) was employed (Lemoine et al. 2019). Bootstrap analysis (FBP + TBE) was performed with 1000 replicates, with all other parameters set to default.

Bayesian Inference (BI) phylogenetic trees based on chloroplast and ITS sequences were constructed using PhyloSuite v1.2.2 (Zhang et al. 2020). Sequences were aligned using the “auto” strategy under normal alignment mode with MAFFT v7.313 (Katoh and Standley 2013) and the resulting files were further adjusted manually using MEGA v11.0 (Tamura et al. 2021). Gblocks (Talavera and Castresana 2007) was applied within PhyloSuite to remove ambiguous sites and gaps. ModelFinder (Kalyaanamoorthy et al. 2017) was used to select the most appropriate evolutionary model. According to the Bayesian Information Criterion (BIC), GTR+F+I+G4 was selected as the optimal model for chloroplast data. Bayesian phylogenetic analysis was performed using MrBayes 3.2.6 (Ronquist et al. 2012) with a partitioned model (two parallel runs, 2,000,000 generations), discarding the first 25% of sampled data as burn-in. For ITS data, SYM+G4 was identified as the best nucleotide evolution model based on BIC. Bayesian phylogenetic inference was conducted using MrBayes 3.2.6 (Ronquist et al. 2012) with a partitioned model (two parallel runs, 10,000,000 generations) and the first 25% of sampled data were discarded as burn-in.

The resulting Maximum Likelihood (ML) and Bayesian Inference (BI) phylogenetic trees were visualised using FigTree v1.4.0.

## ﻿Results

### ﻿Morphological comparison

Figs 2–5, Table 1

**Figure 2.**
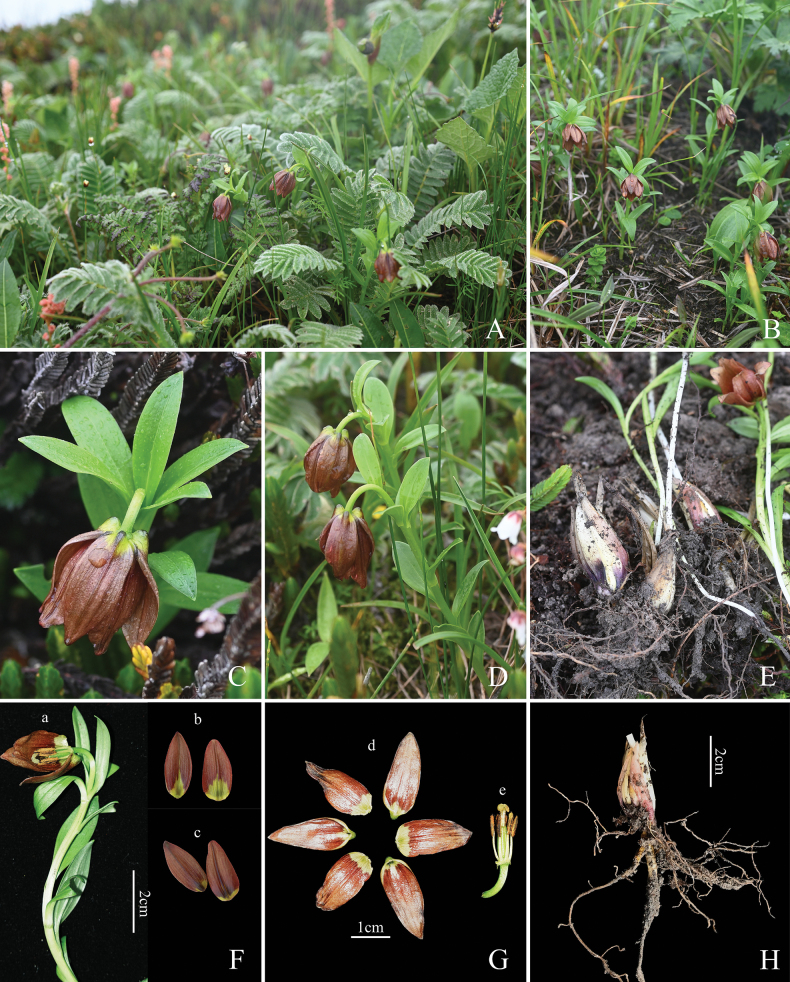
Habitat and morphology of *Liliumbrunneum* T.Wang & Y.D.Gao, sp. nov. **A** habitat **B** habit **C** pedicel **D** leaf **E** bulb **F** transverse section of the flower (a) adaxial surface of the petal (b) abaxial surface of the petal (c) **G** anatomy of the petal (d) pistil and stamen (e) **H** bulb with scale.

**Figure 3.**
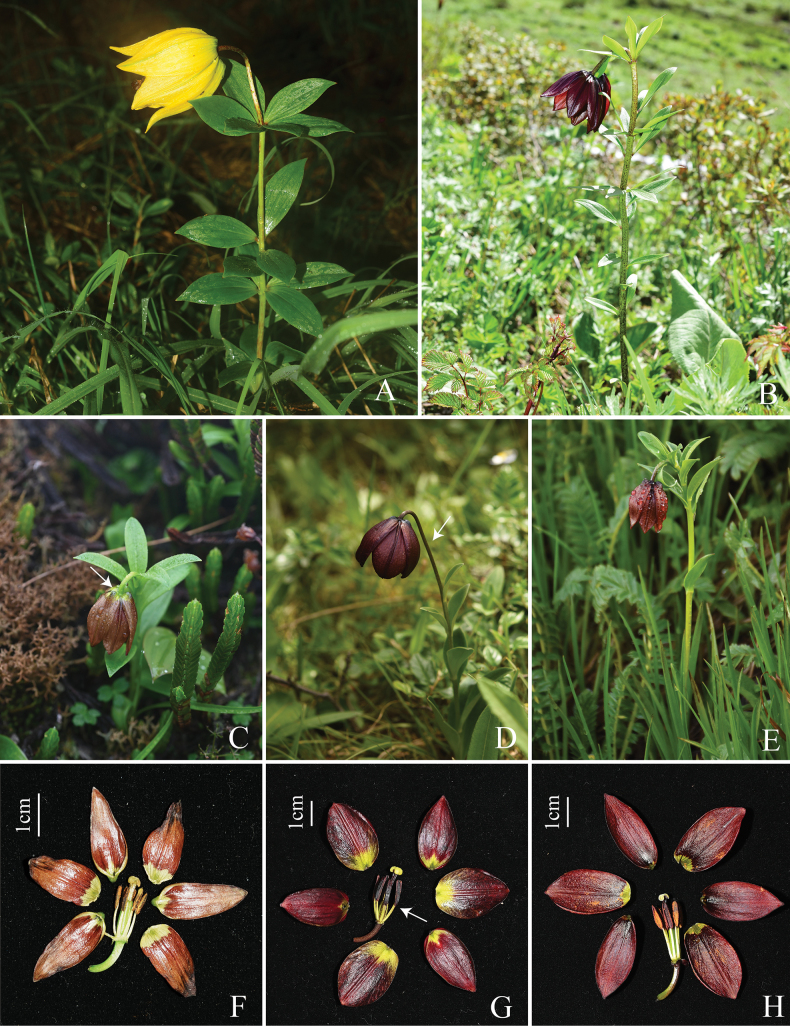
Comparison of several closely related species. **A***Liliummedogense***B***L.paradoxum***C***L.brunneum***D***L.souliei***E***L.saccatum***F** anatomical diagram of the flower of *L.brunneum***G** anatomical diagram of the flower of *L.souliei***H** anatomical diagram of the flower of *L.saccatum*.

*Liliumbrunneum* (Fig. 2) is distinctly different from *L.medogense* (Fig. 3A) and *L.paradoxum* (Fig. 3B), as the latter species are characterised by their taller plants with larger flowers of different coloration, and the possession of whorled foliage (Table 1). In particular, the whorled leaves of the latter two species are distinctive features not found in other members of the *L.souliei* complex. Principal component analysis (PCA) was used to assess the four species of *Lilium* – *L.brunneum* (Fig. 3C), *L.souliei* (Fig. 3D), *L.saccatum* (Fig. 3E) and *L.georgei* (Fig. 5B) – which are most similar to one another.

**Table 1.** Morphological comparisons of the *Lilium* species studied (The table presents the mean values of the measurements, with the corresponding minimum and maximum ranges provided in parentheses).

Characters		*L.brunneum*	*L.medogense*	*L.paradoxum*	*L.souliei*	*L.georgei*	*L.saccatum*
Bulb	length (cm)	3.4 (3.3–3.6)	2.9 (2.6–3.4)	2.5 (1.6–3.4)	2.8 (1.9–3.6)	5.0 (3.8–6.3)	2.9 (2.5–3.2)
	width (cm)	1.5 (1.4–1.6)	2.6 (2.4–2.9)	2.2 (0.9–3.5)	1.5 (1.0–2.4)	3.1 (2.4–3.9)	1.8 (1.4–2.1)
Pedicel	length (cm)	1.6 (0.7–2.6)	4.9 (3.8–6.0)	4.6 (2.3–6.2)	5.1 (2.8–10.5)	3.8 (3.1–4.9)	2.2 (1.8–2.6)
Stem	length (cm)	22.3 (16.6–27.9)	45.6 (37.4–52.1)	40.5 (19.7–66.9)	16.8 (9.5–25.2)	28.1 (18.7–36.5)	24.5 (13.2–40.7)
Leaf	middle leaf length (cm)	3.4 (2.6–4.4)	5.2 (4.7–6.1)	3.8 (2.1–7.0)	4.0 (2.3–6.6)	4.7 (3.0–6.4)	3.0 (1.9–3.8)
	middle leaf width (cm)	0.7 (0.5–1.0)	1.8 (1.5–2.3)	1.2 (0.7–2.1)	0.8 (0.5–1.2)	1.6 (1.2–2.1)	1.0 (0.6–1.2)
	arrangement	scattered	verticillate	verticillate	scattered	scattered	scattered
Flower	corolla width (cm)	2.0 (1.3–3.0)	5.6 (4.3–7.4)	5.5 (3.9–6.8)	4.0 (2.5–5.1)	4.2 (3.8–4.9)	2.5 (1.8–3.1)
	basal colour	brown to light brown	yellow	purple	purple-red	purplish-blue	purple-red
	filaments and ovary	closely appressed	closely appressed	closely appressed	spreading	closely appressed	closely appressed

**Figure 4.**
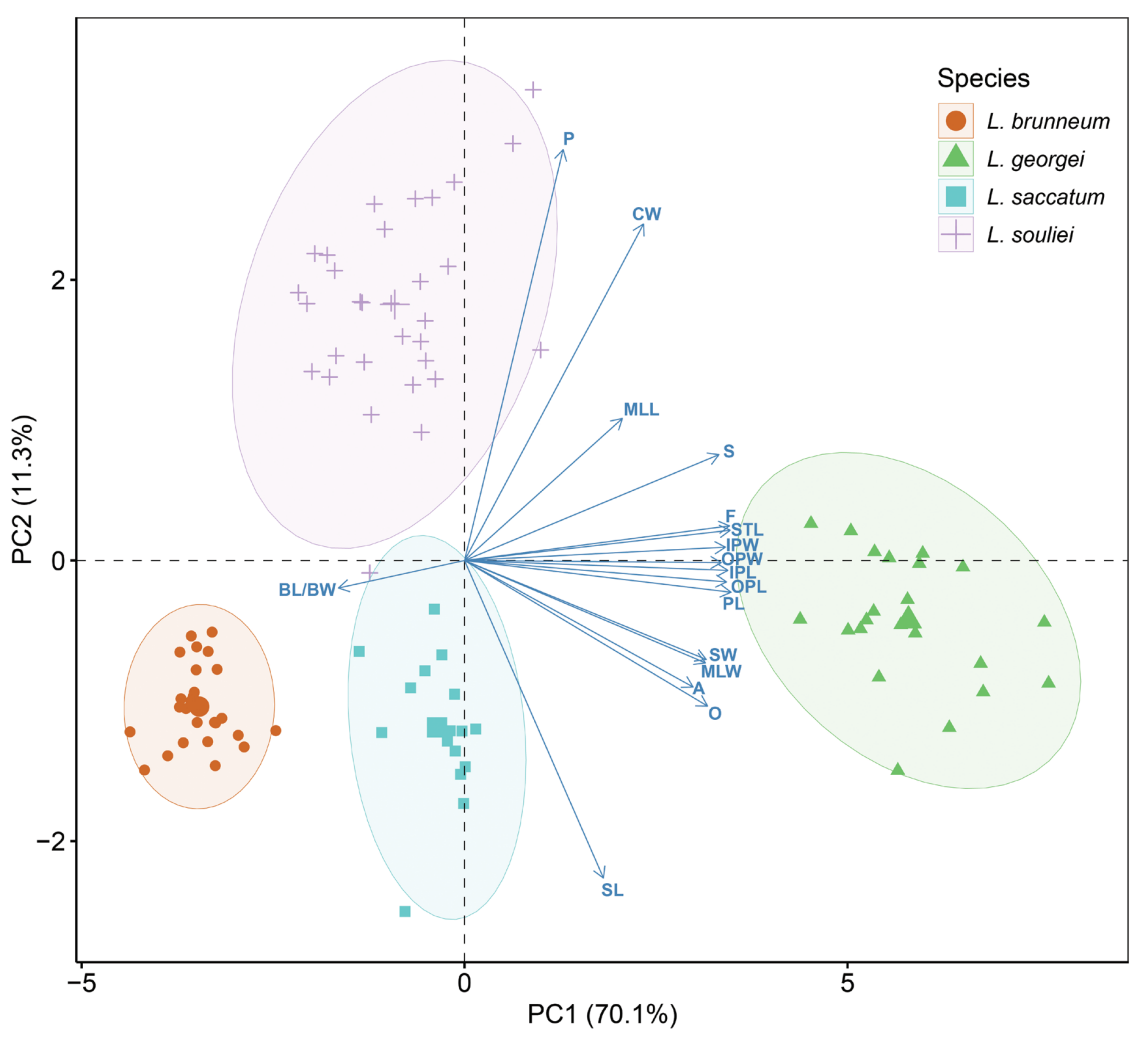
Principal components analysis (PCA) biplot displaying different traits and 96 individuals on PC1 and PC2. For abbreviations and codes of different traits (see Suppl. material 1).

The resulting PCA plot visually illustrates the distribution of the species within the reduced-dimensional space defined by the principal components. The first two components collectively account for 81.4% of the total variance in the dataset (PC1: 70.1%; PC2: 11.3%), demonstrating that this visualisation provides a robust representation of the dataset’s variability (Fig. 4). PC1 is primarily driven by pistil length (PL), stamen length (STL) and filament length (F), while PC2 is predominantly influenced by pedicel length (P) and corolla width (CW). The PCA clearly separates *L.souliei*, *L.saccatum*, *L.georgei* and *L.brunneum* based on the analysed variables, supporting their morphological distinctiveness (Fig. 4).

**Figure 5.**
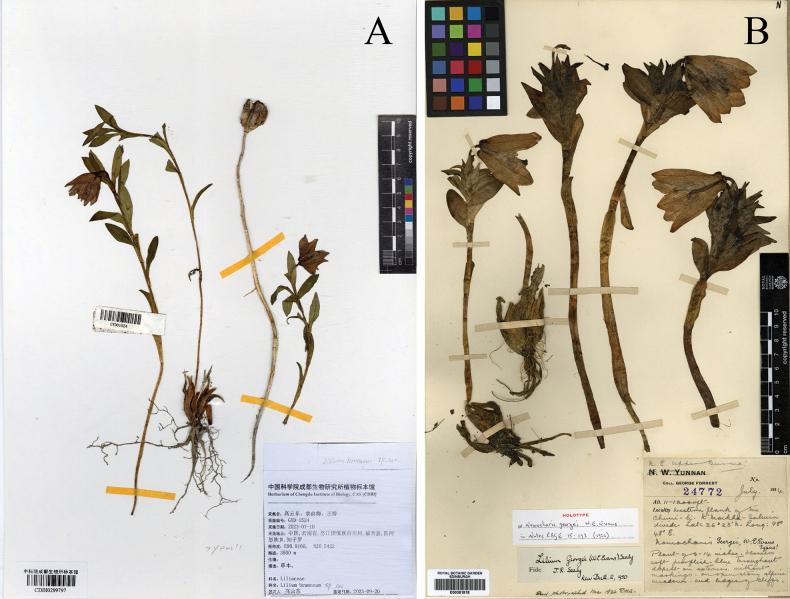
Comparison of specimens of *Liliumbrunneum* and *L.georgei***A** holotype of *L.brunneum* (CDBI0299797) **B** holotype of *L.georgei*. (https://data.rbge.org.uk/herb/E00381818).

### ﻿Phylogenetic analyses

Figs 6, 7

The analysis was based on molecular data, specifically ITS (ITS1, 5.8S and ITS2) sequences and the complete chloroplast genome. We utilised forty-one ITS sequences, with lengths ranging from 610 bp to 633 bp prior to alignment. After alignment correction, the sequence lengths were 647 bp with 245 variable sites and 395 conserved sites. In addition, we analysed thirty-eight complete chloroplast genomes with sequence lengths ranging from 151,083 bp to 152,915 bp before alignment and 158,552 bp after alignment correction, containing 7,643 variable sites and 147,778 conserved sites.

**Figure 6.**
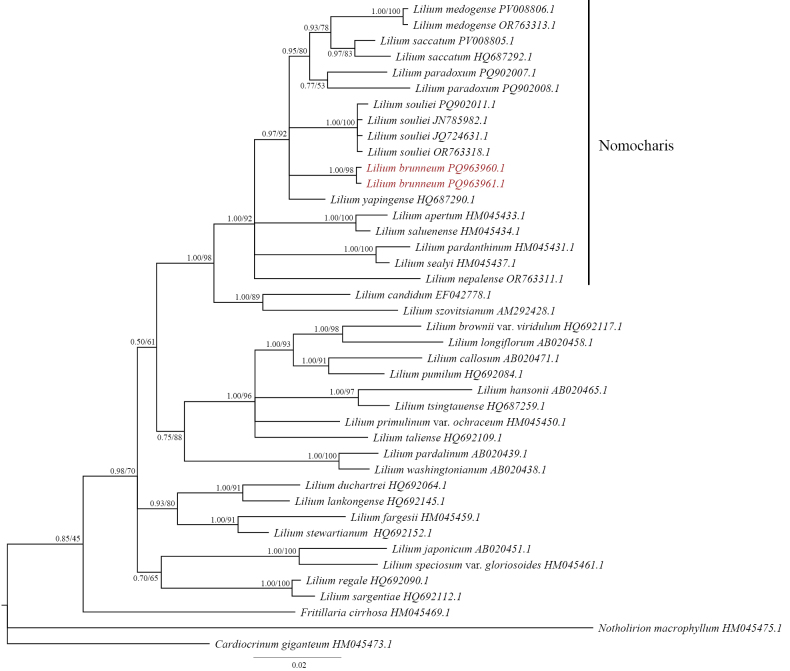
Phylogenetic trees for selected *Lilium* species were constructed based on nuclear ITS sequences using Bayesian Inference (BI) and Maximum Likelihood (ML) methods. The values at the nodes represent Bayesian posterior probabilities (PP) to the left of the slash and bootstrap support values (BS) to the right. The target species is highlighted in red.

**Figure 7.**
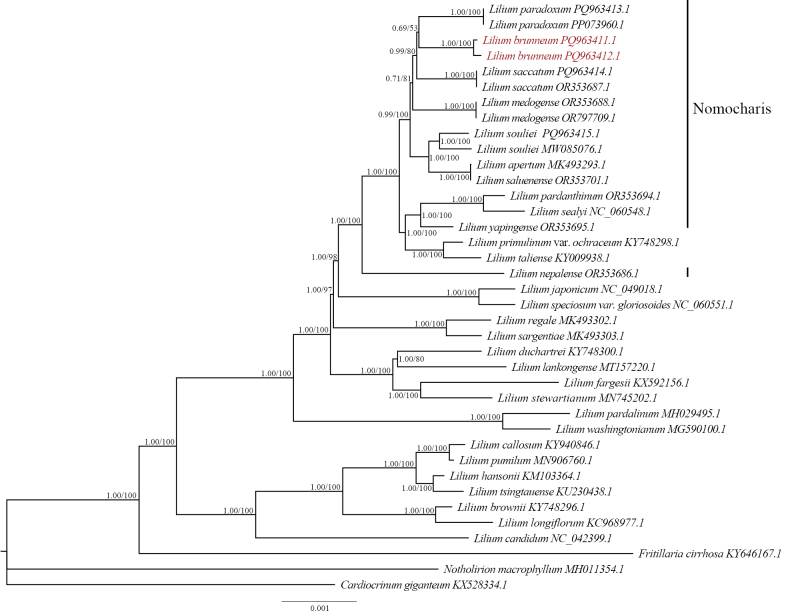
Phylogenetic trees for selected *Lilium* species were constructed based on the complete chloroplast genomes using both Bayesian Inference (BI) and Maximum Likelihood (ML) methods. The numbers at the nodes indicate Bayesian posterior probabilities (PP) to the left of the slash and bootstrap support values (BS) to the right. The target species is highlighted in red.

The Bayesian and Maximum Likelihood (ML) trees derived from both chloroplast and ITS data are largely consistent. In the ITS phylogenetic tree, individuals of *L.brunneum* from the two populations form a distinct clade, which is allied with a clade comprising *L.souliei*, *L.yapingense* and another clade containing *L.paradoxum*, *L.medogense* and *L.saccatum* (Fig. 6). The grouping of the latter three species is strongly supported (PP = 0.95, BS = 80%). All the aforementioned species form a well-supported clade (PP = 0.97, BS = 92%, Fig. 6). In the chloroplast consensus tree, the two *L.brunneum* populations cluster together, forming a sister clade with *L.paradoxum*, with moderate support. This support significantly increases after the inclusion of *L.paradoxum* (PP = 0.99, BS = 80%, Fig. 7). Additionally, all members of the *L.souliei* complex were found to cluster within a single clade, which is highly supported (PP = 0.99, BS = 100%).

### ﻿Taxonomic treatment

#### ﻿ Lilium brunneum

Taxon classificationPlantaeLilialesLiliaceae

T.Wang & Y.D.Gao sp. nov.

4854AAA6-9178-58BC-AE99-18F7C8DD2BB8

urn:lsid:ipni.org:names:77359676-1

Figs 2 , 3 , 4 , 5 and Table 1

##### Type.

China • Yunnan: Fugong county, Gaoligongshan Range [高黎贡山] (on the border with Myanmar); near Zhiziluo [知子罗], Biluoxueshan Range [碧罗雪山], 3500–3800 m. 10 July 2023, *Y.D. Gao GYD1524* (holotype CDBI0299797) (Fig. 5A), 11 July 2023, *GYD1530* (paratype CDBI0299796).

##### Diagnosis.

*Liliumbrunneum* shares morphological similarities with *L.georgei*, *L.souliei*, and *L.saccatum* but exhibits distinct differences that set it apart. Compared to *L.georgei*, *L.brunneum* is characterised by its shorter stature and smaller flowers. In contrast to *L.souliei*, it possesses shorter pedicels, and its filaments are closely appressed to the ovary. When compared to *L.saccatum*, *L.brunneum* is distinguished by its unique petal coloration, which ranges from brown to light brown with a greenish-yellow base. (Table 1, Fig. 2).

##### Description.

Perennial herbs with narrowly ovoid bulbs, 1.4–1.7 cm in diam.; scales white, outermost partially purplish-red, lanceolate. 1.5–3 cm × 6–10 mm. Stem erect, 16–28 cm. Leaves 6–12, scattered, narrowly elliptic, lanceolate, margin sometimes sparsely papillose. Flower solitary, nodding, campanulate. Tepals brown to light brown, usually paler towards base, unspotted, basally gibbous, greenish yellow; outer elliptic, 1.8–2.6 × 0.5–1.1 cm, apex shortly pointed; inner 0.6–1.2 cm wide; nectaries greenish yellow, not papillose. Stamens converging, adnate to the ovary; filaments to 1.0 cm, glabrous, green; anthers dorsifixed at approximately the middle, purple-brown, 4–7 mm. Ovary cylindrical, 7–11 mm long, 2–3 mm wide, green; style shorter than ovary, 5–7 mm; stigma swollen. Capsule subglobose, 1.5–2 cm in diam.

##### Phenology.

Flowering from June to July; fruiting from August to October.

##### Habitat and distribution.

On open stony alpine meadows and edges of bushes. 3500–3800 m. NW Yunnan (Fugong[福贡]) and bordering Myanmar (Kachin).

##### Etymology.

The epithet and Chinese name adopted here both denote the perianth colour of light brown resembling that of caramel.

##### Conservation status and IUCN preliminary assessment.

Through extensive field surveys, we identified two populations of *L.brunneum* located in the Gaoligongshan Range (on the border with Myanmar) and near Zhiziluo in the Biluoxueshan Range. The area of occupancy (AOO) was estimated to be approximately 32 km^2^. Although each population contains more than 300 individuals, the number of mature individuals is fewer than 200. *L.brunneum* grows above the snowline at altitudes exceeding 3500 m. However, ongoing global climate warming may result in a reduction of its habitat area. Based on the criteria of the International Union for Conservation of Nature (IUCN 2024), we recommend classifying *L.brunneum* as an endangered species (EN, B2ab(ii+iii), C2a(i)).

## ﻿Discussion

The overall size of *Liliumbrunneum* is significantly smaller when compared to *L.medogense* and *L.paradoxum*. Based on specimen measurements, the average plant height and corolla width for *L.medogense* are 45.6 cm and 5.6 cm, respectively, while those of *L.paradoxum* are 40.5 cm and 5.5 cm. In contrast, *L.brunneum* displays a distinctly shorter average plant height of 22.3 cm and a corolla width of only 2.0 cm (Table 1, rows 5 and 9). Notably, the former two species can be easily distinguished within the *L.souliei* complex owing to their significantly larger sizes compared to the other members. Furthermore, both species exhibit whorled leaves (typically 3–5 whorls), a characteristic absent in the remaining members of the complex. Specifically, *L.medogense* is distinguished by its bright yellow flowers, making it particularly prominent amongst the complex, as the other members typically present more or less purplish hues. Consequently, these two species were not included in further fine-scale comparisons below, as they cannot be confused with the other members of the complex.

Regarding the remaining members, *L.brunneum* exhibits several distinguishing characteristics when compared to *L.souliei*, *L.saccatum* and *L.georgei*. Principal Component Analysis (PCA) based on morphological measurements from multiple individuals demonstrates clear boundaries between these species (Fig. 4). As a predominant member within the complex, *L.souliei* is positioned in the upper-left quadrant and displays wide morphological variation (Fig. 4), likely associated with its extensive geographic distribution (Fig. 1) facilitating a broad range of variations in response to niche diversity. In comparison to *L.brunneum*, *L.souliei* is characterised by longer pedicels (measured from the flower base to the bract, Fig. 3D), a greater distance between the filaments and ovary (Fig. 3G) and purple-coloured tepals. The PCA plot indicates a complete lack of overlap between *L.brunneum* and *L.georgei* (Fig. 4). Although the collection sites for *L.brunneum* are geographically closest to those of *L.georgei* (Fig. 1), the latter possesses a larger habit, particularly in flower size (Fig. 5B, Table 1), and exhibits more striking colouration, as is noted in the description on the collection label of *L.georgei*, where Forrest wrote: ‘flowers that are soft blue-purple throughout, deepest on the exterior’ (G. Forrest 24772, https://data.rbge.org.uk/herb/E00381818). These differences collectively support the clear delineation of these two species.

While *L.brunneum* is situated closest to *L.saccatum* in the PCA plot, they remain distinctly isolated without any overlap (Fig. 4). The principal components contributing to this separation are primarily flower organ size (e.g. dimensions O). A detailed comparison between *L.saccatum* and *L.brunneum* can also be found in Table 1 and Suppl. material 1. In addition to these differences, *L.brunneum* exhibits lighter-coloured outer tepal bases (Figs 2C, 3C) and features a pale green style (Fig. 3F), whereas the style of *L.saccatum* is dark purplish-black (Fig. 3H). Notably, their geographic distributions are not connected with, but rather widely separated from, that of *L.souliei* (Fig. 1).

The molecular phylogenetic analysis also supports the distinct status of the new species. While the nuclear ITS region has certain limitations due to its relatively short length (approximately 630 bp), it remains sufficient for species delimitation, although it lacks the resolution needed to clarify detailed relationships amongst selected taxa (Yao et al. 2010; Zhang et al. 2022). In the ITS phylogenetic tree, *L.medogense*, *L.saccatum*, *L.paradoxum*, *L.souliei*, *L.brunneum* and *L.yapingense* Y.D. Gao & X.J. He constitute a robustly supported clade (PP = 0.97, BS = 92%, Fig. 6). As previously mentioned, this clade contains several parallel sub-clades due to insufficient informative sites; however, the separation of each recognised species remains clear. Amongst these species, individuals from the two populations of *L.brunneum* form a distinct clade that is allied with *L.souliei*, *L.yapingense* and a clade consisting of *L.paradoxum*, *L.medogense* and *L.saccatum* (Fig. 6). The convergence of the latter three species is supported by considerable evidence (PP = 0.95, BS = 80%), suggesting a closer relationship compared to the other members. The clustering of *L.yapingense* within this clade indicates that this dwarf lily, characterised by its whitish to pinkish campanulate flowers, as described by Gao et al. (2013a), may share a common ancestry with the members of the *L.souliei* complex.

In the chloroplast phylogenetic inferences, the tree topologies generated by Bayesian inference (BI) and maximum likelihood (ML) are congruent. The chloroplast consensus tree demonstrates that the two populations of *L.brunneum* cluster together, forming a sister clade to *L.paradoxum*, with moderate support; this confidence significantly increases upon the inclusion of *L.saccatum* (PP = 0.99, BS = 80%, Fig. 7). Moreover, all members of the *L.souliei* complex are found clustered within the same clade with robust support (PP = 0.99, BS = 100%); however, two species from the former genus *Nomocharis* are also included and positioned as sisters to *L.souliei*. Consequently, under chloroplast phylogenetic analysis, the complex is not monophyletic, revealing incongruence between nuclear ITS and plastid genome data.

According to prior research, such incongruences are common within the genus *Lilium* and the most plausible explanation may involve genetic introgression, with the chloroplast tree providing a more accurate reflection of geographic relationships (Gao et al. 2013b, 2015). For instance, *L.souliei*, which encompasses the broadest distribution range within the complex, overlaps geographically with *L.apertum* Franch. and *L.saluenense* (Balf. f.) S.Y. Liang, which are sister species on the chloroplast tree, receiving strong support (PP = 1.00, BS = 100%). Similarly, although *L.taliense* Franch. is classified within the Sinomartagon-clade in the nuclear ITS phylogeny, it clusters with species from the Nomocharis-clade in the chloroplast tree due to sympatric distributions, likely resulting from introgression via interspecific hybridisation. In fact, *Lilium* species distributed throughout the Hengduan Mountains region have been consistently found clustered within a single clade in both previous and current plastid-based phylogenetic trees (Gao et al. 2015; Duan et al. 2022; Yuan and Gao 2024). This pattern suggests that substantial gene flow has occurred or is ongoing amongst these species within this biodiversity hotspot.

While gene flow can be detected amongst species that are partially sympatric and distributed in the Hengduan Mountains, it is unexpected that several sympatric *Lilium* species exhibit no evidence of gene exchange. For example, the new species coexists with *L.yapingense*, which, according to the nuclear phylogeny, is quite closely related to the entire *L.souliei* complex. However, there is no evidence of interaction between them at the chloroplast genome level, which is unexpected, as introgression would typically be anticipated in such cases. Comparative studies on three gradient-distributed lilies in the same region have shown asymmetric gene flow amongst species, with shared chloroplast genome types (Gao et al. 2020). Although *L.brunneum* is found near to *L.yapingense*, they appear to lack gene exchange, as evidenced by the intact nature of their chloroplast genomes in the plastid phylogenetic tree, a surprising observation considering that they share the same locality and blooming period. The latter aspect has been identified as a critical factor influencing the isolation of gene exchanges amongst lilies (Gao et al. 2020; Feng et al. 2025). This scenario indicates the presence of unknown mechanisms that prevent hybridisation amongst these species, which are likely associated with the high levels of endemism observed in this region.

Further examples of this phenomenon can also be observed within the complex itself. While the distribution ranges of *L.saccatum* and *L.medogense* are geographically proximate (Fig. 1), their phylogenetic relationship is relatively distant. *L.saccatum* thrives in shrubby grasslands on mountain slopes at elevations exceeding 3600 m, whereas *L.medogense* is found in fissured clearings at the edges of alpine wetlands interspersed with fir forests at lower elevations. This ecological disparity may suggest the presence of isolating mechanisms beyond niche diversification and habitat isolation; for instance, differences in floral structures potentially driven by their respective pollinator assemblages (Levin 1978; Baack et al. 2015; Liu et al. 2019) may contribute to the maintenance of species boundaries, thereby restricting gene flow amongst these sympatric lilies and further enhancing biodiversity and endemism within the Hengduan Mountains region.

In conclusion, both morphological and molecular evidence confirm that *L.brunneum* is a distinct new species. This finding enhances our understanding of *L.souliei* and its closely related species, further enriching the catalog of lilies in the Hengduan Mountains region. Additionally, this research contributes to our understanding of species boundaries and the mechanisms contributing to the high levels of endemism observed in this area. Future efforts, including field studies, systematic research with additional data (such as population-level research utilising next-generation sequencing) and analyses of niche diversity, are needed to elucidate the possible undetected species, their origins and the mechanisms maintaining species boundaries.

## Supplementary Material

XML Treatment for Lilium brunneum

## ﻿Additional information

### Author ORCIDs

Ting Wang https://orcid.org/0009-0005-2338-7011

Xiuying Shen https://orcid.org/0009-0002-1560-7270

De Wang https://orcid.org/0009-0007-4767-6904

Ying Zhao https://orcid.org/0009-0008-3800-6949

Xiaomei Qu https://orcid.org/0009-0002-8836-6906

Yundong Gao https://orcid.org/0000-0002-0534-2128

### Data availability

All of the data that support the findings of this study are available in the main text or Supplementary Information.

## Supplementary materials

Supplementary material 1 Measurement data of *Liliumbrunneum*, *Liliummedogense*, *Liliumparadoxum*, *Liliumsouliei*, *Liliumgeorgei*, and *Liliumsaccatum* Data type xlsx https://binary.pensoft.net/file/1298778This dataset is made available under the Open Database License (http://opendatacommons.org/licenses/odbl/1.0/). The Open Database License (ODbL) is a license agreement intended to allow users to freely share, modify, and use this Dataset while maintaining this same freedom for others, provided that the original source and author(s) are credited.Ting Wang, Xiuying Shen, De Wang, Ying Zhao, Xiaomei Qu, Yundong Gao

Supplementary material 2 GenBank accession numbers for sequences utilised in phylogenetic analyses Data type xlsx https://binary.pensoft.net/file/1298779This dataset is made available under the Open Database License (http://opendatacommons.org/licenses/odbl/1.0/). The Open Database License (ODbL) is a license agreement intended to allow users to freely share, modify, and use this Dataset while maintaining this same freedom for others, provided that the original source and author(s) are credited.Ting Wang, Xiuying Shen, De Wang, Ying Zhao, Xiaomei Qu, Yundong Gao
